# Diabetes mellitus is a significant risk factor for the development of liver cirrhosis in chronic hepatitis C patients

**DOI:** 10.1038/s41598-017-09825-7

**Published:** 2017-08-22

**Authors:** Xu Li, Yang Gao, Hongqin Xu, Jie Hou, Pujun Gao

**Affiliations:** 1 Department of Hepatology, The First Hospital of Jilin University, Jilin University, No. 71 Xinmin Street, Changchun, 130021 China; 2 Jilin Province Key Laboratory of Infectious Disease, Laboratory of Molecular Virology, Changchun, 130021 China; 3 Department of Neurology, The First Hospital of Jilin University, Jilin University, No. 71 Xinmin Street, Changchun, 130021 China; 4 Department of Nephrology, The First Hospital of Jilin University, Jilin University, No. 71 Xinmin Street, Changchun, 130021 China

## Abstract

We explored the association between diabetes mellitus (DM) and the risk of hepatitis C virus (HCV)-related liver cirrhosis in Chinese patients with chronic hepatitis C (CHC). To examine the link between DM and liver cirrhosis, we conducted a case-control study of 210 Chinese CHC patients diagnosed with liver cirrhosis, comparing them to an age- and sex-matched control group of 431 CHC patients without liver cirrhosis. We conducted logistic regression analyses adjusting for demographic features and liver cirrhosis risk factors, and found that DM increased the risk of developing liver cirrhosis 2-fold [adjusted odds ratio (AOR), 2.132; 95% confidence interval (CI), 1.344–3.382]. Furthermore, the proportion of liver cirrhosis patients and CHC-only patients with elevated serum triglycerides (>1.8 mmol/L) were 5.2% and 17.4%, respectively, yielding an AOR of 0.264 (95% CI, 0.135–0.517). Multivariate analyses that stratified the risk of developing HCV-related liver cirrhosis in DM patients by gender revealed that the estimated AOR (95% CI) for males was 0.415 (0.178–0.969). In conclusion, DM was associated with an increased risk of developing liver cirrhosis in CHC patients in China. Furthermore, among patients diagnosed with both CHC and DM, females had an increased risk of liver cirrhosis development.

## Introduction

Liver cirrhosis exemplifies end-stage chronic liver disease, and its prevalence is rising worldwide^1, 2^. In China, major known causes of liver cirrhosis include hepatitis B virus (HBV) or hepatitis C virus (HCV) infection and excessive alcohol consumption. In particular, HCV infection is a significant health problem and differs from other hepatitis viruses in that it is a systemic disease, rather than just a liver disorder^3^.

Recently, numerous extrahepatic manifestations of HCV infection have been reported; these include cardiovascular, central nervous system, renal, and metabolic diseases^4^. Among the latter, diabetes mellitus (DM) is common in the general population. Studies that have assessed the association between DM or insulin resistance (IR) and HCV infection clearly demonstrate a significantly higher incidence of DM in patients with chronic HCV than in the general population, and show that HCV is significantly more common in patients with DM^5–7^.

In our previous study, we demonstrated that DM increases hepatocellular carcinoma risk in treatment-naïve chronic hepatitis C (CHC) patients^8^. Here, we conducted a case-control study in which we further investigated the association between DM, specific diabetes-related factors, and liver cirrhosis risk in CHC patients, controlling for other known liver cirrhosis risk factors.

## Results

### Patient demographics and clinical characteristics

Demographic and clinical data of the study participants are summarized in Table 1. We obtained complete diagnostic records for all 641 study participants, of which 431 had a diagnosis of CHC alone (CHC-only patients) and 210 were diagnosed with HCV-related liver cirrhosis (liver cirrhosis patients). The liver cirrhosis group was comprised of 39.5% males, with a median age of 59.00 (53.00, 66.25) years. The control group (CHC-only) was sex- and age-matched to the liver cirrhosis group, with 45.7% males and a median age of 57.00 (52.00, 63.00) years. The two groups exhibited significant differences with regard to demographic characteristics, including the prevalence of DM and gallstones. Notably, DM was significantly more prevalent in liver cirrhosis patients than in CHC-only patients (21.4% vs. 14.4%; *P = *0.025). However, the prevalence of hypertension was not significantly different between the two groups.

**Table 1:** Demographic and clinical characteristics of cases and controls.

Variable	Liver cirrhosis N = 210	CHC-only N = 431	*P*
Male, N (%)	83 (39.5)	197 (45.7)	0.138
Age (years)	59.00 (53.00, 66.25)	57.00 (52.00, 63.00)	0.208
Diabetes, N (%)	45 (21.4)	62 (14.4)	0.025
Hypertension, N (%)	41 (19.5)	112 (26.0)	0.072
Gallstones, N (%)	60 (28.6)	59 (13.7)	<0.001
AST (IU/L)	60.00 (41.00, 92.73)	53.00 (31.00, 94.50)	0.009
ALT (IU/L)	50.00 (30.00, 79.65)	67.00 (30.00, 132.00)	0.002
GGT (IU/L)	47.05 (25.85, 85.40)	53.00 (25.00, 107.50)	0.214
ALP (IU/L)	90.80 (70.98, 124.50)	82.00 (65.00, 108.00)	0.002
TBil (umol/L)	27.40 (18.63, 48.55)	16.00 (11.50, 22.50)	<0.001
ALB (g/L)	31.00 (26.10, 35.73)	38.30 (35.30, 41.50)	<0.001
CHE (IU/L)	3378.50 (2267.50, 4978.00)	7011.00 (5300.00, 8289.00)	<0.001
Triglycerides (mmol/L)	0.86 (0.65, 1.13)	1.14 (0.86, 1.59)	<0.001
Cholesterol (mmol/L)	3.48 (2.84, 4.10)	3.78 (3.28, 4.53)	<0.001
Glucose (mmol/L)	5.27 (4.82, 6.32)	5.29 (4.85, 5.95)	0.729

Continuous variables are expressed as median (25th, 75th percentiles). Categorical variables are displayed as numbers and percentages.

AST = aspartate aminotransferase, ALT = alanine aminotransferase, ALP = alkaline phosphatase, GGT = gamma-glutamyl transpeptidase, TBil = total bilirubin, ALB = albumin, CHE = cholinesterase, CHC = chronic hepatitis C.

Patients in the liver cirrhosis group had elevated levels of AST, ALP, and TBil compared to the CHC-only group. Conversely, the levels of ALT, ALB, CHE, triglycerides, and cholesterol were higher in the CHC-only group compared to the liver cirrhosis group. The two groups had similar glucose and GGT levels.

### Factors associated with liver cirrhosis development in CHC patients

Our univariate analyses suggested a higher incidence of gallstones and diabetes in patients who developed liver cirrhosis (Table 2). However, patients with CHC-only had higher levels of triglycerides (>0.8 mmol/L). In addition, sex, age, diabetes, hypertension, triglycerides, cholesterol, and gallstones were considered for multivariate analysis. After adjusting for potential confounding factors, the independent factors most strongly associated with liver cirrhosis were gallstones and DM. The associated risk of liver cirrhosis was two-fold higher in those with DM [AOR (95% CI), 2.132 (1.344–3.382); *P = *0.001], and two- to three-fold higher in those with gallstones [AOR (95% CI), 2.590 (1.701–3.946); *P* < 0.001].

**Table 2:** Univariate and multivariate analyses of variables associated with hepatitis C virus-related liver cirrhosis.

Variable	Liver cirrhosis N = 210	CHC-only N = 431	*P* ^*#*^	AOR (95% CI)*	P**
Sex			0.138	—	—
Female, N (%)	127 (60.5)	234 (54.3)			
Male, N (%)	83 (39.5)	197 (45.7)			
Age	59.00 (53.00, 66.25)	57.00 (52.00, 63.00)	0.208	—	*—*
Diabetes			0.025	2.132 (1.344–3.382)	0.001
No, N (%)	165 (78.6)	369 (85.6)			
Yes, N (%)	45 (21.4)	62 (14.4)			
Hypertension			0.072	0.595 (0.387–0.915)	0.018
No, N (%)	169 (80.5)	319 (74.0)			
Yes, N (%)	41 (19.5)	112 (26.0)			
Triglycerides			<0.001	0.264 (0.135–0.517)	<0.001
≤1.8 mmol/L, N (%)	199 (94.8)	356 (82.6)			
>1.8 mmol/L, N (%)	11 (5.2)	75 (17.4)			
Cholesterol			0.577	—	—
≤6.0 mmol/L, N (%)	204 (97.1)	415 (96.3)			
>6.0 mmol/L, N (%)	6 (2.9)	16 (2.9)			
Gallstones			<0.001	2.590 (1.701–3.946)	<0.001
No, N (%)	150 (71.4)	372 (86.3)			
Yes, N (%)	60 (28.6)	59 (13.7)			

CHC = chronic hepatitis C; AOR = adjusted odds ratio; CI = confidence interval.

^#^ *P* value for univariate analysis.

*Adjusted for gender, age, hypertension, triglyceride, cholesterol, gallstones, and diabetes

**P value for multivariate analysis.

Interestingly, multivariate analysis also showed significantly different prevalence of hypertension and elevated triglycerides between the liver cirrhosis patients and the controls.

### Association between diabetes duration, treatment, and complications, and the risk of developing liver cirrhosis

Because our studies identified diabetes as a major risk factor associated with liver cirrhosis, we further analyzed the association between liver cirrhosis risk and the following DM-related factors: the length of time over which the patient had DM, DM treatment method, whether or not the patient had DM complications (retinopathy, nephropathy, or neuropathy), and the presence of other health and lifestyle factors. Table 3 summarizes the results of analyses comparing liver cirrhosis and CHC-only patients (controls) in patients with DM. A total of 45 liver cirrhosis patients and 62 controls with current or a history of DM were evaluated. Univariate analyses indicated that factors associated with a greater risk of developing liver cirrhosis in DM patients included lower triglyceride levels (*P = *0.017) and the presence of gallstones (*P = *0.004). Univariate analysis also showed significantly different prevalence of diabetic retinopathy between the liver cirrhosis patients and controls (*P = *0.023).

**Table 3:** Association between the risk of liver cirrhosis development and diabetes duration, diabetes treatment, diabetes complications, and other variables in diabetes patients.

Variables	Liver cirrhosis N = 45	CHC-only N = 62	*P* ^*#*^	AOR (95% CI)*	P**
Sex			0.065	0.415 (0.178–0.969)	0.042
Female, N (%)	27 (60.0)	26 (41.9)			
Male, N (%)	18 (40.0)	36 (58.1)			
Age			0.554	—	—
<50	6 (13.3)	6 (9.7)			
≥50	39 (86.7)	56 (90.3)			
Duration of diabetes			0.873	—	-
≤5 years, N (%)	34 (75.6)	46 (74.2)			
>5 years, N (%)	11 (24.4)	16 (25.8)			
Diabetes treatment			0.836	—	—
No, N (%)	22 (48.9)	30 (48.4)			
single drug, N (%)	23 (51.1)	31 (50.0)			
multiple drugs, N (%)	0	1 (1.6)			
Diabetic retinopathy			0.023	—	—
No, N (%)	44 (97.8)	52 (83.9)			
Yes, N (%)	1 (2.2)	10 (16.1)			
Diabetic neuropathy			1.000	—	—
No, N (%)	45 (100.0)	61 (98.4)			
Yes, N (%)	0 (0.0)	1 (1.6)			
Diabetic nephropathy			0.571	—	—
No, N (%)	43 (95.6)	61 (98.4)			
Yes, N (%)	2 (4.4)	1 (1.6)			
Triglycerides			0.017	0.268 (0.079–0.910)	0.035
≤1.8 mmol/L, N (%)	41 (91.1)	45 (72.6)			
>1.8 mmol/L, N (%)	4 (8.9)	17 (27.4)			
Cholesterol			1.000	—	—
≤6.0 mmol/L, N (%)	42 (93.3)	58 (93.5)			
>6.0 mmol/L, N (%)	3 (6.7)	4 (6.5)			
Gallstones			0.004	4.938 (1.524–15.998)	0.008
No, N (%)	32 (71.1)	57 (91.9)			
Yes, N (%)	13 (28.9)	5 (8.1)			

CHC = chronic hepatitis C; AOR = adjusted odds ratio; CI = confidence interval.

Continuous variables are expressed as median (25th, 75th percentiles).

^#^ *P* value for univariate analysis.

*Adjusted for sex, age, duration of diabetes, diabetes treatment, diabetic retinopathy, diabetic neuropathy, diabetic nephropathy, triglycerides, cholesterol, gallstones, and liver cirrhosis.

We also performed multivariate analyses examining the roles of sex, age, duration of DM, treatment for DM, DM retinopathy, DM nephropathy, DM neuropathy, triglycerides, cholesterol, and gallstones. Male CHC patients were at lower risk for liver cirrhosis development (AOR, 0.415; 95% CI, 0.178–0.969; *P = *0.042). Significant differences were observed in the prevalence of reduced triglyceride levels and gallstones between liver cirrhosis patients and the controls (*P = *0.035; *P = *0.008).

Conversely, our multivariate analyses did not indicate that the length of time over which CHC patients had diabetes affected the risk of liver cirrhosis development. Moreover, we found no correlation between the risk of developing liver cirrhosis and the DM treatment method or whether or not the patient had DM complications (retinopathy, nephropathy, or neuropathy).

## Discussion

In line with previous findings^3, 9^, our study demonstrated a two-fold higher prevalence of DM in cirrhosis patients, compared to a CHC-only control group that was age- and gender-matched. Likewise, Miyaaki *et al*. compared the pathological and clinical characteristics of steatosis and metabolic syndrome in CHC patients, in order to identify risk factors for CHC with severe fibrosis^10^. They found that DM was significantly associated with severe fibrosis. Similarly, a cohort study revealed that DM was an independent predictor of disease progression in CHC patients^11^.

As we know, IR and DM negatively affect the liver in patients with chronic HCV^12^. These metabolic effects are associated with hepatic steatosis development^13^. Fibrosis progresses more rapidly to cirrhosis in patients with HCV, IR, and T2DM, except for HCV genotype 3, which is less responsive to treatment with interferon (IFN)^14, 15^. IR is independently associated with the progression of fibrosis^16^ and negatively influences the effects of HCV on the liver and its treatment. In addition, insulin is a growth factor and the high levels of insulin in patients with IR lead to adverse outcomes in patients with chronic HCV. Furthermore, insulin stimulates the secretion of matrix proteins and other precursors of hepatic fibrosis by hepatic stellate cells^17^. Insulin interferes with the intracellular downstream antiviral effects of IFN to decrease both the rapid and sustained virological responses^14, 15, 18^.

In this study, we found that females had a higher risk of liver cirrhosis in CHC patients who also had DM. The reason for this might be as follows: firstly, we know that the HCV life cycle depends on host cell cholesterol metabolism, which is disrupted by HCV core protein and the nonstructural protein 5A^19^. HCV causes impaired lipid export, impaired lipid degradation, and enhanced lipogenesis, which lead to hepatic steatosis and consequent liver injury^19, 20^. Furthermore, IR, which is a common feature in DM, could also lead to the accumulation of triglycerides by increasing the flux of free fatty acids into the liver^21^. We hypothesize that the above mentioned pathways by which HCV leads to liver injury might be enhanced when patients have concomitant DM, and the degree of enhancement differs in men and women. Secondly, DM is a risk factor for NAFLD, which can lead to liver fibrosis. Although the epidemiology of gender differences in NAFLD is somewhat controversial, data from cross-sectional studies^22–32^, which are based primarily on a histological diagnosis of nonalcoholic steatohepatitis (NASH), tend to suggest that the risk of NASH and advanced fibrosis is higher in females than males (independent of metabolic factors)^22, 26–28^, with only a few studies reporting conflicting results^23, 25, 29^.

Additionally, we found differences in triglyceride levels in CHC and liver cirrhosis patients. One possible explanation is that patients might change their dietary intake because of abdominal distension or other symptoms accompanying liver cirrhosis, which could reduce the serum levels of triglycerides. Furthermore, we found a higher prevalence of gallstones in patients with liver cirrhosis than in those with CHC alone, which is in line with the results of previous studies^33–36^. This may be attributed to the pathogenesis of gallstones in patients with chronic liver disease, which includes changes in the composition of hepatic bile, as well as gallbladder hypomotility^34, 37^. Gallbladder motility is decreased by increased levels of female sex steroidal hormones, which usually occur with cirrhosis. Furthermore, liver cirrhosis patients have thicker gallbladder walls than chronic hepatitis-only patients, and these thicker walls may contribute to gallbladder hypomobility^38–40^.

Notably, we found no evidence that treatment for DM conferred an increased risk for the development of liver cirrhosis. The reasons might be as follows: first, drugs for DM treatment usually include metformin and insulin. Metformin could reduce the levels of circulating insulin^41–44^ and aid in improving responses to antiviral treatment, including a combination of pegylated IFN alpha-2a (PEG-IFNα-2a) and ribavirin (RBV), in patients with CHC of the naïve genotype 1 (G1) subtype^45^. Therefore, metformin might decrease the incidence of liver cirrhosis in patients with CHC. However, external insulin could increase the levels of circulating insulin and aggravate liver fibrosis, as discussed above. Therefore, hypoglycemic drugs might have different effects on liver cirrhosis development in different CHC patients, resulting in the lack of an observable impact of DM treatment on liver cirrhosis development in this study.

The principal limitations of this study are its retrospective design, and the lack of details regarding the type of oral hypoglycemic agents used for treatment. Further study is needed to understand the association between hypoglycemic agents and liver cirrhosis development. Secondly, the number of cases in our study was not large, leading to a small number of subjects in the subgroup analyses. The case numbers were limited by our desire to exclude patients without blood lipid and glucose data. Therefore, our insignificant findings with respect to diabetes duration, treatment, and complications may be a reflection of these low numbers.

In conclusion, we found that DM increased the risk of liver cirrhosis development among CHC patients in China. Our findings also suggested that, for patients dually diagnosed with CHC and DM, female patients may experience an enhanced risk of developing liver cirrhosis.

## Methods

### Patient selection

This was a cross-sectional study to investigate risk factors associated with liver cirrhosis in CHC patients who were hospitalized at The First Hospital of Jilin University in China from January 2011 to December 2016. All methods were carried out in accordance with the approved guidelines. In total, 641 patients with CHC infection, as diagnosed by the presence of HCV RNA and anti-HCV antibodies in the serum for ≥6 months, were recruited for inclusion in our study. Of these, 210 patients had liver cirrhosis. After matching for sex and age in the cases group, 431 patients constituted the control sample group.

Subjects were excluded for the following criteria: (i) co-infection with HBV or human immunodeficiency virus; (ii) history or evidence of any type of cancer; (iii) history or evidence of infection with other hepatitis types; or (iv) presence of other liver disease, such as nonalcoholic fatty liver disease (NAFLD) or alcoholic liver disease.

The Independent Institutional Review Board of The First Hospital of Jilin University approved the study protocol and the recruitment of human subjects. We obtained written informed consent from each patient upon enrollment in the study.

### Diagnosis of liver cirrhosis

Liver cirrhosis was diagnosed either by liver biopsy, or based on combined clinical findings, biochemistry, and radiology.

### Diagnosis of DM

DM was diagnosed in patients with known history of DM under anti-diabetic therapy or at least one of the following criteria: (1) fasting glucose level ≥7.0 mmol/L; (2) random glucose level ≥11.1 mmol/L; or (3) 2-hpost-load plasma glucose ≥11.1 mmol/L^46^.

### Study variables

We analyzed the following demographic, lifestyle, and health-related variables in this study: sex, age, hypertension, gallstones, presence of DM, duration of DM, treatment of DM, and complications of DM (such as diabetic retinopathy, nephropathy, or neuropathy). Furthermore, we examined the following biochemical parameters: alkaline phosphatase (ALP), alanine transaminase (ALT), aspartate aminotransferase (AST), total bilirubin (TBil), albumin (ALB), cholinesterase (CHE), gamma-glutamyl transferase (GGT), triglycerides, cholesterol, and glucose.

### Statistical analysis

Continuous variables are presented as the median (25th, 75th percentiles), and categorical variables are displayed as numbers and percentages. To determine the significance of our findings, we employed a chi-square test for categorical variables. For normally distributed continuous variables, we employed an independent sample *t*-test. All tests were two-tailed. We employed multivariate logistic regression to adjust for possible confounding effects among the variables. Additionally, adjusted odds ratios (AORs) and 95% confidence intervals (CIs) were calculated for these comparisons. Statistical analyses were performed using SPSS version 13.0 software (SPSS Inc., Chicago, IL, USA). *P*-values < 0.05 were considered to represent statistical significance.
